# Spatiotemporal Analysis of the 2014 Ebola Epidemic in West Africa

**DOI:** 10.1371/journal.pcbi.1005210

**Published:** 2016-12-08

**Authors:** Jantien A. Backer, Jacco Wallinga

**Affiliations:** 1 Centre for Infectious Disease Control, National Institute for Public Health and the Environment, Bilthoven, The Netherlands; 2 Department of Medical Statistics and BioInformatics, Leiden University Medical Center, Leiden, The Netherlands; Johns Hopkins Bloomberg School of Public Health, UNITED STATES

## Abstract

In 2014–2016, Guinea, Sierra Leone and Liberia in West Africa experienced the largest and longest Ebola epidemic since the discovery of the virus in 1976. During the epidemic, incidence data were collected and published at increasing resolution. To monitor the epidemic as it spread within and between districts, we develop an analysis method that exploits the full spatiotemporal resolution of the data by combining a local model for time-varying effective reproduction numbers with a gravity-type model for spatial dispersion of the infection. We test this method in simulations and apply it to the weekly incidences of confirmed and probable cases per district up to June 2015, as reported by the World Health Organization. Our results indicate that, of the newly infected cases, only a small percentage, between 4% and 10%, migrates to another district, and a minority of these migrants, between 0% and 23%, leave their country. The epidemics in the three countries are found to be similar in estimated effective reproduction numbers, and in the probability of importing infection into a district. The countries might have played different roles in cross-border transmissions, although a sensitivity analysis suggests that this could also be related to underreporting. The spatiotemporal analysis method can exploit available longitudinal incidence data at different geographical locations to monitor local epidemics, determine the extent of spatial spread, reveal the contribution of local and imported cases, and identify sources of introductions in uninfected areas. With good quality data on incidence, this data-driven method can help to effectively control emerging infections.

## Author Summary

Infectious disease modelling has become an established tool to inform decisions in infection control. For outbreaks that are confined to one geographical location, modelling approaches exist to analyse epidemic data, monitor infection incidence and assess the effect of control measures. For outbreaks that are spread over various adjacent districts or countries, such as the recent Ebola epidemic in West Africa, few modelling approaches are available that can analyse the spatiotemporal data. To study the epidemic spread within and between districts and countries, we have developed an analysis method that uses the full resolution of these data. The outbreak is represented as a network of local epidemics that are interconnected through travellers that are infected in one district, but observed to be infected in another. The main advantages of this method are that it needs little data and does not make strong assumptions on parameter values; a disadvantage is that it does not take underreporting into account. The spatiotemporal method can monitor the development of local epidemics, determine the extent of spatial spread, reveal the contribution of local and imported cases, and identify sources of introductions in uninfected areas. These results can help to effectively control emerging infections.

## Introduction

Over the last decades, infectious disease modelling has become an established tool to assess the effect of control measures and to inform infection control. Such analysis methods have for instance been applied to local outbreaks of SARS [1], Chikungunya [2] and MERS CoV [3]. However, when an outbreak is distributed over a larger area, the spatial aggregation of epidemic data can result in time series that are too coarse to adequately capture the disease dynamics [4]. Ideally, the analysis should allow for the higher spatial resolution of the data [5]. The recent Ebola epidemic provides such a case in which extended spatiotemporal data were collected.

In 2014, West Africa was heavily affected by an epidemic of Zaire Ebola virus that was unprecedented in its magnitude [6]. Earlier epidemics in the east and middle of the African continent were usually confined to a limited area and brought under control within a few months [7]. Similarly, the West African epidemic began in a rural area around Guéckédou, Guinea at the end of 2013, but started to deviate from the typical pattern when cases were observed first in Guéckédou City and later in the large urban area of capital Conakry. Around the same time the infection spread from the border area around Guéckédou into Liberia, followed by Sierra Leone two months later. Their capitals Monrovia and Freetown were infected shortly after these incursions, allowing the virus to spread in highly urbanised populations with limited health care facilities. In July 2014, the WHO recognised the Ebola epidemic as a public health emergency of international concern. While aid programs were being extended and implemented, the epidemic reached its peak in the autumn of 2014, when hundreds of cases were reported weekly. Subsequently, incidence rates started to decline, bringing the epidemic gradually under control. All countries were declared free of Ebola at some point in 2015, but transmission seems to be ongoing at a low level as evidenced by the sporadic observation of new cases.

From early on in the epidemic, infectious disease models were applied to inform public health officials tasked with disease control. Notably, they aimed to estimate the effective reproduction number *R*_*e*_ that expresses the expected number of individuals infected by a typical case. As such, it is an intuitive measure to assess whether the epidemic is under control (*R*_*e*_ < 1) or not (*R*_*e*_ > 1). The earliest models used—for lack of better data—sparse and incomplete incidences, aggregated by the whole region [8, 9], by country [6, 10–13] or by a single district [14]. A special mention needs to be made of Gire et al. [15] and the WHO emergency response team [6] who shared their data with the scientific community, which sparked a myriad of research efforts while the epidemic was ongoing.

Data aggregation by country, however, is too coarse to follow how an epidemic develops within a country. For instance, in Liberia and Sierra Leone, all districts were affected, while in Guinea, six districts seemed to have escaped infection. Also, Conakry did not experience an exponentially growing outbreak like Freetown and Monrovia did, despite their comparable population size and density. These differences between and within countries are evident from the district-level incidence data that the WHO published weekly from November 2014 onwards. Using the full resolution of the data could potentially reveal useful insights into the epidemic spread within and between districts. Such an analysis should take into account that an observed case in one district could have been infected in another. For instance, Yang et al. [16] analysed the epidemic in the 14 districts of Sierra Leone using an ensemble adjustment Kalman filter, but because they ignored the possible role of Guinea and Liberia, independent introductions from other sources needed to be allowed.

Here, we aim to develop a method to analyse epidemics for which longitudinal incidence data at different geographical locations are available. We build on a method for estimating time-varying reproduction numbers [17, 18] that tracks the effective reproduction number over time. For a single infected population, the model is fully specified by the incidence data and the serial interval distribution, i.e. the time between observations of paired cases. To allow for multiple populations, this model needs to be extended. We assume that a fraction of newly infected persons leave their district and will be observed in another district. This dispersal follows a gravity-type power law [19]: infectees scatter over districts depending on the distance to and the population size of the destination. In this way, the full epidemic can be treated as an interconnected network of local epidemics that are separated in space and time. Analysis of the epidemic with this method not only provides estimates for the local effective reproduction numbers over time, but also determines the contribution of each location to the epidemic. It should be noted though that this is a descriptive rather than a predictive method.

**Fig 1:**
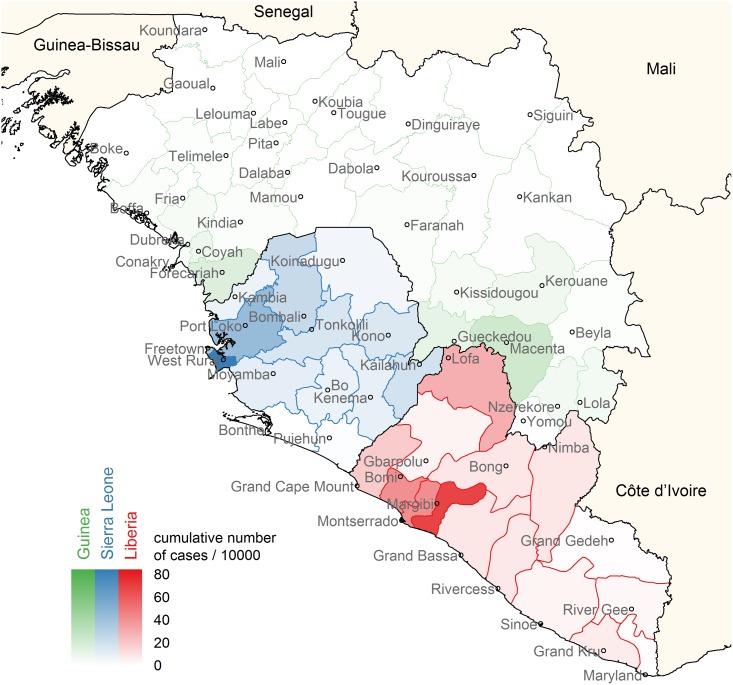
Cumulative incidence map of Guinea, Sierra Leone and Liberia. Cumulative number of cases per 10000 people in each district of Guinea (green), Sierra Leone (blue) and Liberia (red), over the period December 2013—June 2015. For each district, the location of the most populous city is indicated on the map and used in the analysis. These incidences are the averages over ten augmented data sets to include suspected, probable and confirmed cases.

The spatiotemporal model is applied to the Ebola epidemic in the three most heavily affected countries, assuming a single introduction in Guéckédou, Guinea and taking subsequent human-to-human transmissions within and between countries into account. The weekly incidences of confirmed and probable cases in the patient database and situation reports up to June 2015 are used, as reported by the WHO (data in S1 Data). Incidence data on district level are augmented to include suspected cases for which only data on country level are available (Fig 1 and Methods). In the analysis, we distinguish between countries to be able to account for possible differences between within-country and cross-border transmissions. The effective reproduction numbers and the distribution between local and imported cases are studied for each district, as well as the contribution of different districts to new introductions. Finally, we assess how sensitive the results are to inaccuracies in data and parameters.

## Results

Included in the spatiotemporal analysis of the Ebola epidemic are 33 districts in Guinea, 14 districts in Sierra Leone and 15 districts in Liberia, that differ in cumulative incidence over the study period of December 2013 to June 2015 (Fig 1). Model parameters that describe the local epidemics and the spatial dispersion between districts, are estimated in a Bayesian MCMC framework.

**Fig 2:**
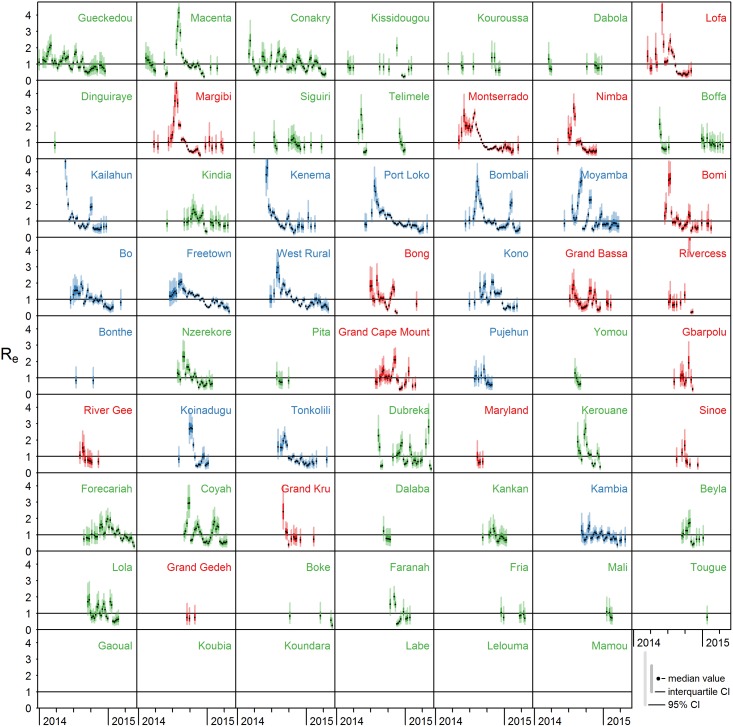
Estimated effective reproduction numbers over time per district. Effective reproduction numbers *R*_*e*_ in all districts of Guinea (green), Sierra Leone (blue) and Liberia (red) over time; median values (black dots), interquartile credible interval (dark coloured bars), and 95% credible interval (light coloured bars). Districts are ordered by time of first observed case; in six districts in Guinea not a single case has been observed.

The local epidemics are captured by the effective reproduction number that is estimated for each district for each week with observed cases (Fig 2). For districts with small outbreaks or fluctuating incidence rates, these numbers vary around the threshold value of *R*_*e*_ = 1, but for districts with large outbreaks, such as Freetown and Port Loko in Sierra Leone and Montserrado in Liberia, the effective reproduction numbers are above one—typically around two—for a prolonged period early in the local epidemic. When little information is available, the posterior distribution of the effective reproduction number reflects the prior distribution. This is the case at the end of a local epidemic, e.g. in Macenta, Guinea, or when few cases are observed in a district, e.g. in Dinguiraye, Guinea. In general, there do not seem to be notable differences between the three countries.

The spatial dispersion parameters are assumed to be constant over time and are estimated for each country, or for each country pair in the case of cross-border transmissions. Only a minority of newly infected people leave for another district, the percentages being similar for the three countries: 8 (95% credible interval: 6–10)% for Guinea, 6 (4–9)% for Sierra Leone and 6 (4–9)% for Liberia. According to the spatial parameter estimates, these people are more attracted to larger cities, and they are less likely to travel long distances. Moreover, they are more likely to stay within the country than to cross a border to a neighbouring country. More details and posterior distributions are given in S1 Text, section 2.

**Fig 3:**
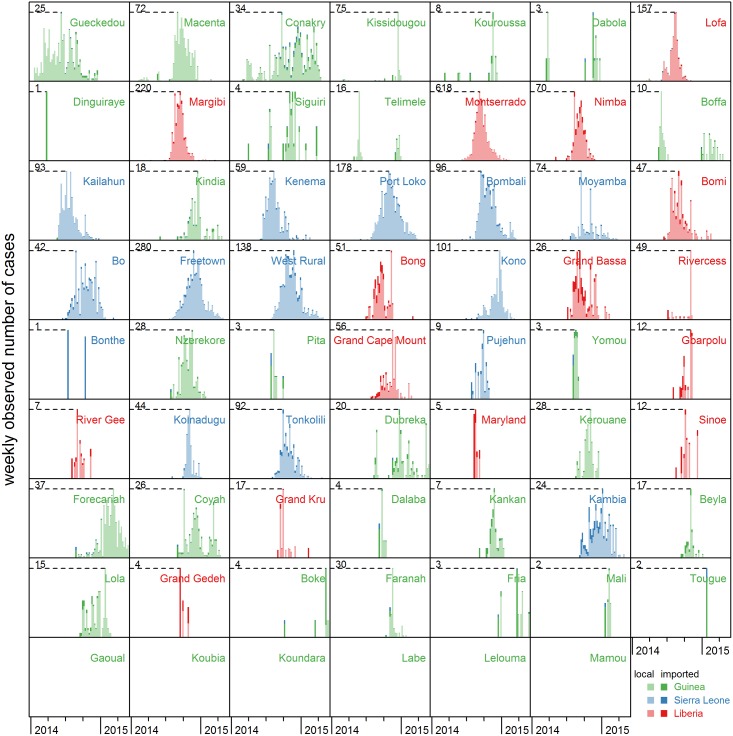
Observed incidence over time per district, classified by origin of cases. Weekly observed incidence in all districts of Guinea (green), Sierra Leone (blue) and Liberia (red), based on median posterior values averaged over ten augmented data sets. A distinction is made between locally generated cases (lightly coloured bar), imported cases from another district in the same country (dark tip of same colour), and imported cases from another country (dark tip of different colour). Districts are ordered by time of first observed case; in six districts in Guinea not a single case has been observed. Note that the y-axis scale depends on the maximal number of observed cases per week in a district, ranging from 1 (Dinguiraye, Guinea) to 618 (Montserrado, Liberia).

By combining the posterior median values of the model parameters that describe local generation of cases (*R*_*e*_) and those that describe the spatial dispersal, we can determine which part of the weekly observed cases is imported from other districts. Most of the observed cases are due to local transmission, i.e. generated in the same district (Fig 3). Although the fractions of generated cases that were exported are found to be comparable for each country, the fractions of observed cases that were imported can differ from district to district. This mainly depends on the incidences in surrounding districts. For instance, the many imported cases in Kambia, Sierra Leone, are the spill-over of the local epidemic in nearby Port Loko. In general, most of the imported cases come from a district in the same country (Fig 3). Of the migrating cases in each country 91 (77–96)% stays in Guinea, 87 (77–100)% stays in Sierra Leone and 100 (88–100)% stays in Liberia. Obviously, cross-border transmissions are much rarer; over the full epidemic 27 (11–69) cross-border transmissions are estimated from Guinea, 110 (1.1 ⋅ 10^-4^ − 221) from Sierra Leone and 0.025 (1.4 ⋅ 10^-5^ − 86) from Liberia. These estimated cross-border cases can for instance be seen in the incidence pattern in Conakry, Guinea, where a considerable amount of imported cases during the later stages of the epidemic is attributed to Sierra Leone.

Instead of studying all imported cases, we now focus on those that have established a new introduction in previously uninfected districts, or a reintroduction in districts without observed cases in the preceding 8 weeks. In total, 69 of such (re)introductions were observed, associated with 136 imported cases. The majority of these occured in Guinea, because it has the largest number of districts and experienced most of the reintroductions. On the other hand, six Guinean districts escaped infection altogether. Even so, the susceptibility of districts to be infected does not seem to differ between countries (survival analysis in S1 Text, section 3).

**Fig 4:**
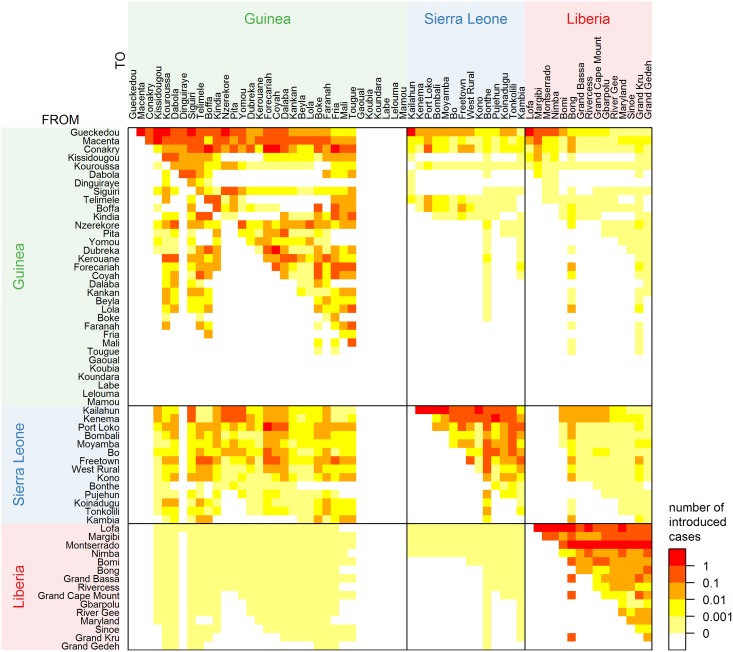
Number of introduced cases per district distributed over possible origin districts, based on median posterior values averaged over ten augmented data sets. Districts are ordered per country by time of first observed case. District columns add up to the total number of observed introduced cases in that district, which can be higher than 1 due to multiple introductions and due to multiple cases per introduction. Colours indicate distinct categories: 1 or more introduced cases (red), between 0.1 and 1 (dark orange), between 0.01 and 0.1 (orange), between 0.001 and 0.01 (yellow), between 0 and 0.001 (light yellow), and 0 (white). The latter category means that this introduction is impossible, because the destination was never infected or the source was not infected at the time of introduction in the destination.

For each introduction event, a probability distribution of the origin is determined, summarised in Fig 4. Introductions in a district are only possible by districts that were infectious at that time; these district pairs are indicated by a coloured square in Fig 4. As the districts are ordered by time of first observed case, most of these district pairs are in the upper triangular part of each country matrix. District pairs in the lower triangular part indicate that reintroductions in previously infected districts are possibly caused by districts that were later infected. Most introductions originate from districts that were infected early in the epidemic in the border area, i.e. Guéckédou and Macenta in Guinea, Kailahun and Kenema in Sierra Leone, and Lofa in Liberia. Once infected, capital cities Conakry in Guinea and Montserrado in Liberia continue to contribute over the course of the epidemic. Despite their large outbreaks, Freetown and West Rural in Sierra Leone play a smaller role in infecting other districts, because they were infected relatively late in the epidemic when most districts were already infected. As observed for the contributions of different districts (Fig 3), most introductions occur within the same country. Guinea, and specifically Guéckédou, also contributes to introductions in Sierra Leone and Liberia by necessity, because the epidemic origin lies in Guinea. Sierra Leone seems to contribute most to cross-border introductions, mainly in Guinea and some in Liberia. On the other hand, the contribution of Liberia to introductions in other countries, is negligible.

**Fig 5:**
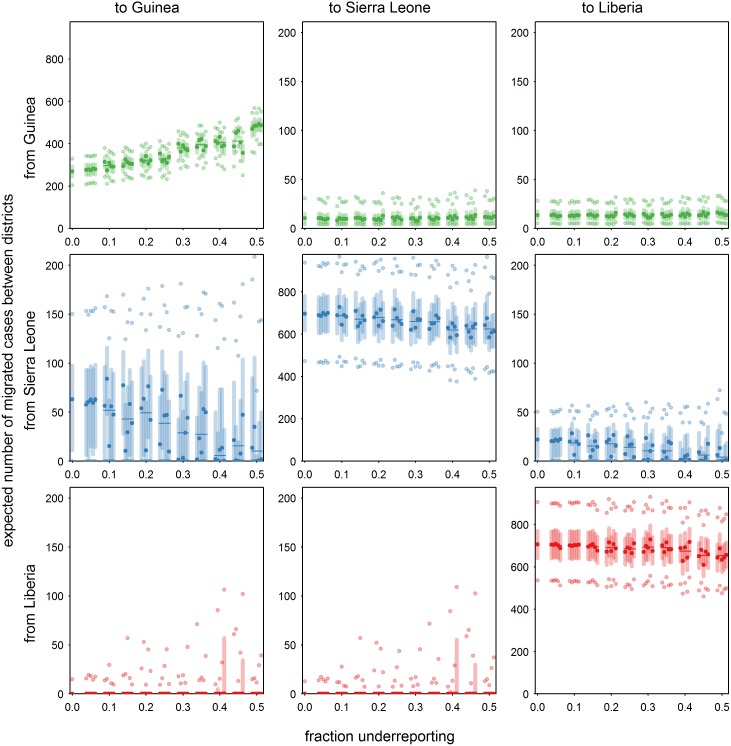
Expected number of migrated cases between districts as a function of underreporting fraction in Guinea, based on median posterior values, with five repetitions per underreporting level, for Guinea (green), Sierra Leone (blue) and Liberia (red). Median value (dark symbol), median value averaged over five repetitions (dark line), interquartile range (light line), and 2.5% and 97.5% percentiles (light symbols).

Our results suggest that Sierra Leone played a large role in (re)introductions in Guinea. However, these introductions may also have been caused by unobserved cases in Guinea because of underreporting. The effect of underreporting is explored in a sensitivity analysis with a focus on Guinea. The underreporting fraction is varied from 0% (perfect reporting) to 50% (actual number of cases is twice the number of reported cases [20]), to explore how it affects the number of migrated cases between countries (Fig 5). In general, the results become more variable when underreporting increases, but two trends are notable. First, the number of migrations within Guinea increases, due to the larger number of cases, while the migration fraction in Guinea decreases from 8.1% to 6.7% (Fig. E in S1 Text, section 4). Secondly, the number of cases from Sierra Leone to Guinea on average decreases by a factor six. In fact, the contribution of Sierra Leone to introductions in Guinea all but disappears (Fig. F in S1 Text, section 4). Only for the transmission from Port Loko, Sierra Leone to Forécariah, Guinea considerable support remains.

The average serial interval is assumed to be 15.3 days, as reported by the WHO emergency response team [6]. In a sensitivity analysis (full results in S1 Text, section 5), the average serial interval is varied from 10 to 16.1 days [21]. With a shorter average serial interval, the migration fractions in Sierra Leone and Liberia are found to be higher which leads to more migrating cases. In Sierra Leone, a large part of these additional migrations leave for other countries, while in Liberia, they stay within the country. This enhanced role of Sierra Leone in cross-border transmissions should however not be overinterpreted with the sensitivity analysis results for underreporting in mind.

## Discussion

We have introduced a method to analyse epidemics for which longitudinal incidence data at different geographical locations is available. This spatiotemporal method combines a model for time-varying effective reproduction numbers in one location [17, 18] with spatial dispersion to other locations [19]. Through this combination, it is possible to quantify between-district transmissions and identify dominant contributors and the relative role of different districts and countries. Our results suggest that, in Liberia, mainly within-country transmissions occurred, while Sierra Leone seems to have contributed considerably to new introductions in neighbouring Guinea. Some evidence of these cross-border transmissions has been provided by phylogenetics and transmission chains. A phylogenetic analysis of sequence data of viruses from all affected countries [22] showed that the virus (lineage B) was reintroduced from Sierra Leone into Guinea. On the other hand, a similar analysis with more Liberian sequences [23] revealed that multiple introductions from Liberia to Guinea occurred in the later stages of the epidemic. An analysis of transmission chains [24] also identified a transmission occurrence from Sierra Leone to Conakry in Guinea. In the same study, the first introduction in Conakry was traced back to Dabola. Our model, however, identified Guéckédou as most likely origin because the first case in Conakry preceded the first case in Dabola by two weeks in the district case data.

Because of the data-driven modelling of the epidemic spread, the results depend strongly on the input, both on the data and on the parameters. Although the weekly incidence data of probable and confirmed cases were augmented to include suspected cases, we assume that these data are complete and that the reporting weeks are free of error. If underreporting were constant over time and space, the estimated parameters are expected to be robust. Heterogeneities in reporting over space would only affect the spatial dispersion parameters, while heterogeneities in reporting over time would also affect the reproduction numbers. The effect of underreporting in Guinea is explored in a sensitivity analysis, choosing a maximal underreporting fraction of 50%, which was used by the WHO for planning the emergency response [20]. For different situations, this fraction is either too high [25] or should be even higher [26]. The sensitivity analysis shows that, in particular, the cross-border transmissions from Sierra Leone are affected, but other cross-border and within-country transmissions as well as the model parameters are fairly robust. Taking underreporting properly into account is only possible if additional independent data are collected to quantify the extent of underreporting, such as phylogenetic information [27] or data on case fatality rates [28].

The assumed serial interval distribution is shown to affect our results, but the level of confidence in this parameter is quite high. In the analysis, an average serial interval of 15.3 days is assumed, based on data of 92 patients of the West Africa outbreak [6], while other estimates [21] are based on less detailed data from different outbreaks. More recently, the WHO emergency response team reported an adjusted average serial interval of 14.2 days [29], based on data of 305 patients. Our sensitivity analysis shows that this new value supports the results. The serial interval distribution is discretised in weeks in the analysis using weekly incidence data. Using daily incidence data—if available—would allow a serial interval distribution in days to be used, diminishing the effect of discretisation.

Also for the spatiotemporal model itself, some model choices and simplifying assumptions have been made. Firstly, the spatial dispersion is modelled by a power-law gravity model. This model is preferred over an exponential decay model with non-dimensionless parameters that are difficult to compare between countries, and over a transmission kernel as is often used in livestock diseases [30] that would require estimating two parameters instead of one. Any spatial dispersion model would fit in the current framework, but one should beware of overparametrisation. Secondly, the model requires that the infections in a district are only caused by cases that are observed in that district. In other words, infected persons may travel in their latent period, but only start infecting other people at their destination. For Ebola this can be a plausible assumption, as infectiousness increases with more severe travel-impeding symptoms, but for other diseases this might not be the case. Thirdly, the model considers an introduction consisting of multiple cases as independent introductions, whereas it is often more likely that they are connected. For instance, the first introduction in Grand Gedeh, Liberia consists of four introduced cases (Fig 3) that might well have been one family. Finally, we have chosen to estimate time-independent spatial parameters. The extent of spatial dispersal might have decreased over the course of the epidemic as a result of control measures such as border control. This could be taken into account by specifying the spatial parameters as a function of time or in different control phases. In a first exploratory analysis with two control phases per country, we use as change points 1 August 2014 for Guinea, 21 August 2014 for Liberia, and 21 September 2014 for Sierra Leone. Results (S1 Text, section 6) indeed indicate that spatial dispersal tends to be smaller and more local in the later stages of the epidemic. As most districts were infected in the earlier stages of the epidemic, these results are hardly surprising and not necessarily an effect of control measures. Regardless, the roles of countries in cross-border transmissions are not markedly different from the original analysis with time-independent parameters.

The main advantages of the method proposed here are that it avoids strong modelling assumptions and requires only the observed case incidence data. Such data are in general collected from the start of the epidemic and are often readily shared. In comparison, data on genetic sequences of pathogens may take longer to become available. The number of estimated parameters are kept to a minimum, and do not need strong priors. No underlying assumptions are made on how the reproduction numbers develop over time, instead they are estimated for each district in each week. In this way the effective reproduction numbers can reveal structural changes in transmission due to control or behavioural changes, and it can capture fluctuating incidence data, e.g. in Conakry, Guinea. Also, the migration fractions are independent of the extent of the spatial dispersal. This allows for the situation where few people migrate, but when they do, they do so over large distances, as has, for instance, been estimated for Liberia. The method by Yang et al. [16] does not make this distinction, but includes the home district (at zero distance) in the migration matrix. This may explain why they find a lower proportion of local transmissions (weighted average of 82%) than we do (weighted average of 94%).

By combining a model for estimating time-varying reproduction numbers with spatial dispersion, we have developed a method that can monitor an epidemic as it spreads within and between districts. As such, it is a descriptive rather than a predictive method. It can however determine the extent of spatial spread, reveal the contribution of local and imported cases, and identify sources of introductions in uninfected areas. Not restricted to Ebola, the method could be applied to spatiotemporal data of any newly emerging disease where little to no immunity exists beforehand in the affected populations, and for which a reasonable estimate for the serial interval distribution exists. This type of analysis can also be applied during an ongoing outbreak, provided that reporting delays are properly taken into account [31]. Monitoring the local epidemics together with developments in neighbouring districts can help allocate public health resources, and pinpointing most likely sources of infection could help make decisions on movement bans or border closures. In this way, spatiotemporal analyses can be instrumental in effectively controlling emerging infections.

## Methods

### Model

The method to analyse spatiotemporal incidence data combines a model for estimating time-varying reproduction numbers [17, 18] and a gravity model for spatial dispersion [19]. A key parameter of epidemics is the effective reproduction number *R*_*e*_ that denotes the expected number of individuals infected by a typical case. As such, it is an intuitive measure to assess whether the epidemic is under control (*R*_*e*_ < 1) or not (*R*_*e*_ > 1). These reproduction numbers can be tracked through time, using knowledge of the serial interval distribution, i.e. the period between the time of disease onset of an infector and the time of disease onset of its infectee. Here we will assume that the time interval between reporting of infector and infectee can be approximated by the serial interval.

The number of cases that are locally generated, or local incidence, Λ_*i*_(*t*) in district *i* at time *t* is informed by the observed incidences *I*_*i*_ in that district in the preceding weeks, through the serial interval distribution *ω* [32]:

$$\Lambda_{i}\left(t\right)=R_{i}\left(t\right)\sum_{\tau =1}^{T}\omega \left(\tau \right)I_{i}\left(t-\tau \right),$$ (1)

where *T* is the cut-off period for the serial interval distribution and *R*_*i*_(*t*) is the reproduction number in district *i* at time *t* at which secondary cases are observed. This instantaneous reproduction number immediately reflects changes in transmission as opposed to the effective or case reproduction number *R*_*e*,*i*_(*t*) that is assigned to the time at which the primary cases are observed [18]:

$$R_{e,i}\left(t\right)=\sum_{\tau =1}^{T}R_{i}\left(t+\tau \right)\omega \left(\tau \right).$$ (2)

Estimating *R*_*i*_(*t*) instead of *R*_*e*,*i*_(*t*) has the advantage that the convolution in Eq 1 only needs to be calculated once. Were district *i* a closed system, without any migrations in or out, the local incidence Λ_*i*_(*t*) would be the expected number of observed cases in district *i* in week *t*. To allow for migrations, the model is extended.

In this framework, local epidemics are connected through movements of infected persons. The observed weekly incidence in each district is a combined effect of locally infected persons and infected persons that have moved in from other districts. Part of the local incidence Λ_*i*_(*t*) generated in district *i* will be observed elsewhere because of migrations. This (country-specific) fraction *f* of persons infected in district *i* scatter over other districts *j* weighted by a dispersion distribution *m*_*i*,*j*_. The expectation of the observed incidence *I*_*i*_(*t*) in district *i* at time *t* is then a summation of the non-migrating local incidence and the incidence caused by migrants from other districts:

$$E\left(I_{i}\left(t\right)\right)=\left(1-f\left(c_{i}\right)\right)\Lambda_{i}\left(t\right)+\sum_{j\neq i}f\left(c_{j}\right)m_{j,i}\Lambda_{j}\left(t\right),$$ (3)

with *c*_*i*_ indicating the country of district *i*. The observed incidence *I*_*i*_(*t*) is assumed to be Poisson distributed [32]. The dispersion term *m*_*i*,*j*_ distributes the migrants over the other districts following a gravity-type model [19]:

$$m_{i,j}=\frac{N_{j}^{\alpha }D_{i,j}^{-\delta (c_{i},c_{j})}}{\sum_{j\neq i}N_{j}^{\alpha }D_{i,j}^{-\delta (c_{i},c_{j})}}.$$ (4)

The dependency on the population size *N*_*j*_ of destination *j* is estimated by exponent *α*, assumed to be the same for all countries. When the probability of migrating to another district is proportional to its size, *α* will be one 1; values of *α* > 1 demonstrate the additional attraction of larger population sizes. The dependency on the distance *D*_*i*,*j*_ between districts *i* and *j* is estimated by exponent *δ*. A value of zero indicates random dispersal, and a value of 2 suggests homogeneous dispersal. The exponent *δ*(*c*_*i*_, *c*_*j*_) describes within-country dispersal when *c*_*i*_ = *c*_*j*_ and between-country dispersal when *c*_*i*_ ≠ *c*_*j*_. A different exponent could be estimated for each pair of countries, but here the cross-border terms from a specific country are identical, *δ*(*c*_*i*_, *c*_*j*_) = *δ*(*c*_*i*_, *c*_*k*_) for *c*_*i*_ ≠ *c*_*j*_ ≠ *c*_*k*_. This means only a distinction is made between whether a migrating infected person stays within the country or leaves for another country.

### Data

The model described above is used to analyse the weekly incidences of probable and confirmed Ebola cases in the patient database up to June 2015, as reported by the WHO. For the 33 districts in Guinea, the 14 districts in Sierra Leone and the 15 districts in Liberia, the number of observed cases per week are given. All districts in Liberia and Sierra Leone reported cases at some stage of the epidemic, but 6 of the 33 districts in Guinea escaped infection all together. The cumulative number of cases in the data set amount to 3642 in Guinea, 11317 in Sierra Leone and 4994 in Liberia. The official case numbers (including suspected cases) aggregated by country on 31 May 2015, however, are 3652 in Guinea, 12827 in Sierra Leone and 10666 in Liberia [33], which shows that about half of the cases in Liberia were never confirmed, leading to a large underrepresentation in the district-level data. This is corrected for by adding the missing cases—10 in Guinea, 1510 in Sierra Leone and 5672 in Liberia—to the data on district level. For each missing case, an observation week and a district is assigned which is sampled proportionally to the weekly incidence data in the districts (code in S1 Code). This is repeated ten times, to generate ten augmented data sets that are analysed separately.

The population size of each district is estimated from the latest available census data [34]. With most recent census data being determined in 2014 for Guinea, in 2008 for Liberia and in 2004 for Sierra Leone, the population size of each district was proportionally adjusted to add up to the total population sizes in 2013 of 11.75 million for Guinea, 4.294 million for Liberia and 6.092 million for Sierra Leone [35]. The location of each district is pinpointed at the location of the administrative centre as this is usually the most populous urban area. Great-circle distances between these locations are calculated by the haversine formula. The country, prefecture, administrative centre, location coordinates, population size and observed weekly incidences for each district are provided in S1 Data.

The WHO [6] estimated that the serial interval distribution for the 2014 Ebola epidemic had a mean of 15.3 days and a standard deviation of 9.3 days. We use a shifted gamma distribution with these parameters, shifted by 1 week [32] and truncated at 8 weeks (i.e. weekly probabilities of 0.3655, 0.3526, 0.1499, 0.0700, 0.0336, 0.0164, 0.0080, 0.0040 for 1 to 8 weeks between observed cases). The truncation of 8 weeks is based on the WHO definition (http://www.who.int/csr/disease/ebola/declaration-ebola-end/en/) that a country is declared free of disease 6 weeks after the last patient has recovered, which is on average 2 weeks after symptom onset [6]. Incidence gaps of more than 8 weeks within a district occur more often in Guinea (11 times) than in Liberia (twice) or Sierra Leone (once) and are treated as separate introductions.

### Analysis

The analysis method is tested on simulated epidemics in randomly generated populations in a single country with 20 districts. The simulation parameters are recovered reasonably well, but in less than 95% of the simulations the true value is captured by the 95% credible interval. This is because the reproduction numbers *R* absorb some of the stochasticity in the data, leading to higher precision and lower accuracy. For this reason, vaguely informative priors are preferred to restrict the reproduction numbers to plausible values. Results and more details are provided in S1 Text, section 1, and R-code in S2 Code.

The model parameters are estimated in a Bayesian MCMC analysis. For the reproduction numbers *R*, a vaguely informative gamma prior is used with mean 1 and shape parameter 2. For the fractions *f*_*c*_ of migrating infected persons, a beta distribution is used with a mean of 0.10 (Beta(1, 9)), and for the parameters *α* and *δ* of the dispersal distribution, uniform priors between -1 and 6 are used, to include the possibility of no population or distance dependence. The analysis is run with 10 chains of length 5000, for each augmented data set; convergence is assessed by eye. The model is analysed in JAGS using R [36], for which the R-code is provided in S1 Code.

For the sensitivity analyses, one of the augmented data sets is used that best resembles the average. For the underreporting sensitivity analysis, a number of cases (depending on the underreporting level) is added to the incidence data of Guinea in the same way as has been done for the augmented data sets. This is repeated five times for each underreporting level. For the serial interval sensitivity analysis, the average serial interval is varied while the shape parameter is kept constant.

## Supporting Information

S1 Text Supporting text. Supporting text containing details of simulated epidemics, posterior distributions of model parameters, survival analysis for infecting districts, sensitivity analysis for underreporting, sensitivity analysis for serial interval, and analysis with time-dependent parameters.
(PDF)

S1 Data Data file. Data for each district comprising country, prefecture, administrative centre, location coordinates, population size and observed weekly incidences, used in analysis.
(TXT)

S1 Code R-code analysis. R-code including JAGS code used for spatiotemporal analysis of the 2014 Ebola epidemic in West Africa.
(R) Click here for additional data file.

S2 Code R-code simulations. R-code for generating random population, simulating epidemic and analysing incidence data, used for testing spatiotemporal analysis method.
(R) Click here for additional data file.
