# Supplementary material for: Spatiotemporal Analysis of the 2014 Ebola Epidemic in West Africa
Source: PLoS Comput Biol. 2016 Dec 8;12(12):e1005210. doi: 10.1371/journal.pcbi.1005210 (PMC5145133; doi:10.1371/journal.pcbi.1005210)
Supplement: S1 Text — Supporting text containing details of simulated epidemics, posterior distributions of model parameters, survival analysis for infecting districts, sensitivity analysis for underreporting, sensitivity analysis for serial interval, and analysis with time-dependent parameters. (PDF) [file pcbi.1005210.s001.pdf]

Supplemental information to
Spatiotemporal analysis of the 2014 Ebola epidemic in West Africa

Jantien A. Backer[1,*], Jacco Wallinga[1, 2]

1 Centre for Infectious Disease Control, National Institute for Public Health and the Environment, The Netherlands

2 Department of Medical Statistics and BioInformatics, Leiden University Medical Center, The Netherlands

*** jantien.backer@rivm.nl**

1 Simulated epidemics

To test the analysis method outlined in the main article, it is applied to simulated epidemics in randomly generated populations. The estimated parameters are compared with the true values to test bias and variance of the estimators.

For each simulated epidemic, a population is generated in a square area. A number of cities is uniformly distributed over the area, and each city is assigned a population size according to a lognormal distribution. An epidemic is started in the city that is closest to the centre by introducing one infected person. Subsequent infections are simulated according to the serial interval distribution and the instantanteous reproduction number R, and distributed over the different cities, according to the population distances and the distribution parameters f, δ and α. The local epidemic in an infected city will start off with an instantanteous reproduction number $R > 1$, but when a certain number of infected persons is reached, it will drop to $R < 1$. This breakpoint is different for each city, according to a lognormal distribution, leading to different final sizes for the local epidemics. To mimic the situation of Ebola, these breakpoints are chosen much lower than the population sizes, bringing the epidemic under control long before depletion of susceptibles comes into effect.

Table A shows the parameter values used in the simulations. The R-code for generating a population, simulating an epidemic and analysing the incidence data are given in supplement

input	value/distribution
number of cities	20
population size per city	LogNormal(10, 1)
serial interval distribution	(0.3700, 0.3569, 0.1517, 0.0708, 0.0340, 0.0166)*
reproduction number R before breakpoint	2
reproduction number R after breakpoint	0.5
breakpoint per city	LogNormal(5, 0.2)
migration fraction f	0.06
distance dependency δ	2.5
population size dependency α	1.3

Table A: True values of model parameters used in simulations.
* weekly probabilities truncated at 6 weeks for ease of computation (truncation of 8 weeks used in Ebola analysis)

S2 Code.

Two hundred epidemics are simulated in this way, with at least 200 infected people to condition on large epidemics. Per simulated epidemic, 5013 (1794 - 5889, median and 95% interval) people are infected in 19 (9-20) of the 20 cities. As the interplay between migration fraction f and reproduction number R affects the parameter estimates, the simulations are analysed with two different sets of priors: a uniform set and a vaguely informative set. The distance dependency δ and population size dependency α have uniform priors in both sets. Priors and averaged posteriors are shown in Tab. B and posterior distributions for each simulation are shown in Fig. A.

The true values used in simulating the epidemics are reasonably well recovered when averaging over all simulations. The informative prior set leads to the smallest mean squared error for the parameters of primary interest, i.e. α, δ and f. The uniform prior set overestimates the reproduction numbers R_{before} and R_{after}, while the informative set pushes them towards the prior mean of one. To assess how these parameter estimates affect the interpretation of the results, the percentage of correctly identified infectors is determined for each simulation. With the uniform prior set, this percentage is 69 (45-94)%, while with the informative prior set, a marginally higher percentage of 70 (45-100)% of the infectors are correctly identified.

In general, the true values are poorly covered by the credible intervals of individual simulations, especially for the migration fraction f with uniform priors. The reason that credible intervals are not wider is because the reproduction numbers R are allowed to vary over a wide range, independent of reproduction numbers in preceding or following weeks. This absorbs

some of the stochasticity in the data, leading to higher precision and lower accuracy. Assuming a profile for R over time (e.g. a step function or S-curve in this case) could give better results, but this cannot be assumed in general. Instead, the slightly informative prior is preferred to restrict R to plausible values.

		uniform priors			informative priors		
	true	posterior	MSE	cov	posterior	MSE	cov
α	1.3	1.3 (0.76-1.8)	0.14	87	1.1 (0.74-1.6)	0.11	76
δ	2.5	2.9 (2.0-3.8)	0.48	82	2.8 (2.1-3.5)	0.38	79
f	0.060	0.051 (0.034-0.071)	0.00085	56	0.066 (0.048-0.085)	0.00039	78
R_{before}	2.0	2.4 (0.53-16)	0.13	100	1.5 (0.27-3.2)	0.25	100
R_{after}	0.50	0.75 (0.12-9.3)	0.065	100	0.65 (0.16-2.3)	0.022	100

Table B: Posterior of model parameters for two sets of prior distributions used to analyse 200 simulated epidemics. The reported posteriors are the means of the median posterior value and the 95% credible intervals of the simulations. The mean squared error (MSE) is based on the posterior medians and the true value. The coverage (cov) is the percentage of simulations that include the true value in their 95% credible intervals. Prior distributions for fraction f are Beta(1,1) for the uniform set and Beta(1,9) for the informative set; prior distributions for reproduction number R are U(0,20) for the uniform set and Gamma(2,2) for the informative set; prior distributions for α and δ are U(-1,6) for both sets. The posterior estimates for reproduction numbers R_{before} and R_{after} are based on all cities in all weeks with observed incidence before and after the breakpoint was reached.

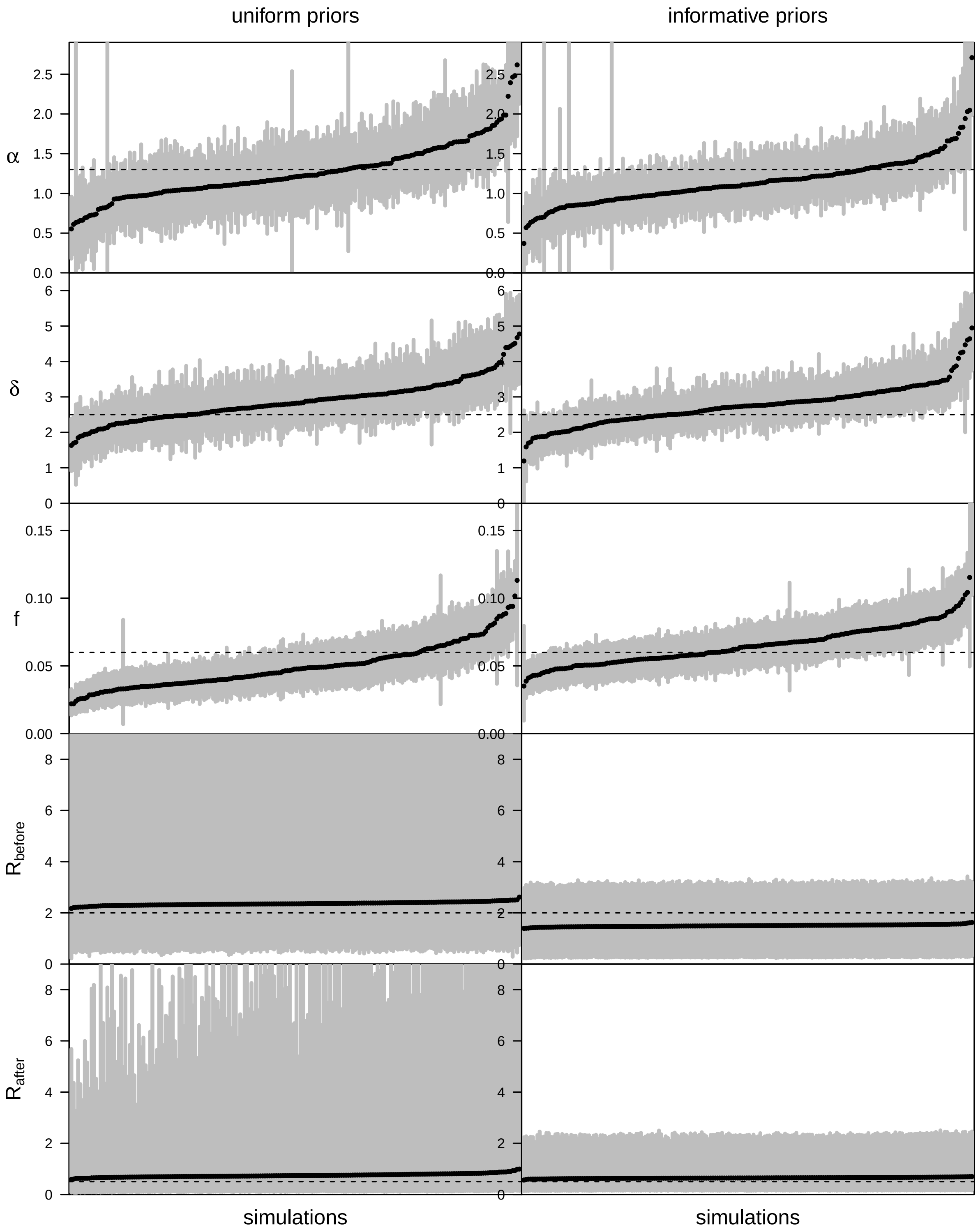

Figure A: Ranked posterior values of population size dependency α, distance dependency δ, migration fraction f, and reproduction numbers R_{before} and R_{after} for 200 simulated epidemics, analysed with uniform priors (left) or informative priors (right). Black dots: median posterior values, grey bars: 95% credible interval, dashed line: true value. The posterior estimates for reproduction numbers R_{before} and R_{after} are based on all cities in all weeks with observed incidence before and after the breakpoint was reached.

2 Posterior distributions of spatial dispersal model parameters

Figure B and Table C summarise the posterior distributions of the three model parameters that drive the spatial dispersal. From the posterior distributions of the individual data sets, it is clear that the results for Liberia are most variable, as the Liberian incidence data needed to be most severely augmented. Even so, they are markedly different from the other countries' posterior distributions. The overall posteriors are therefore believed to capture the differences between countries as well as the uncertainty in the data.

The migration fraction f of newly infected persons that leave their district is estimated for each country (Fig. B). As would be expected, only a small percentage leaves their district. The migration fractions for the different countries are similar, with a slightly larger fraction for Guinea.

Parameter α is a measure of the dependency on the population size of the destination, and is estimated for all countries together (Fig. B). For a value of $\alpha = 1$, migrating persons would choose a destination proportionally to its size, so a twice as big city would attract twice as many people. That the estimated value for α is larger than one - although not significantly - shows the more than proportionally attraction of larger cities.

For the distance dependencies δ, a distinction is made between transmissions within a country and cross-border transmissions from that country (Fig. B). A value of $\delta = 0$ means random dispersal, i.e. independent of how far people need to travel, while for increasing values of δ migrating persons will choose nearer destinations. The results show that long-distance transmissions are most important within Liberia and least important within Sierra Leone, but these values might also be influenced by the specific geographies of the countries. We can however compare the probability of a migrant to travel to a district in the same country (within-country transmission) or in another country (cross-border transmission). For both Guinea and Sierra Leone, the estimated values for cross-border transmission are larger than for within-country transmission. This signifies that a transmission at a certain distance is less likely if the migrant also has to cross a border. For cross-border transmissions from Liberia little data is available, reflected by the recovery of the prior distribution.

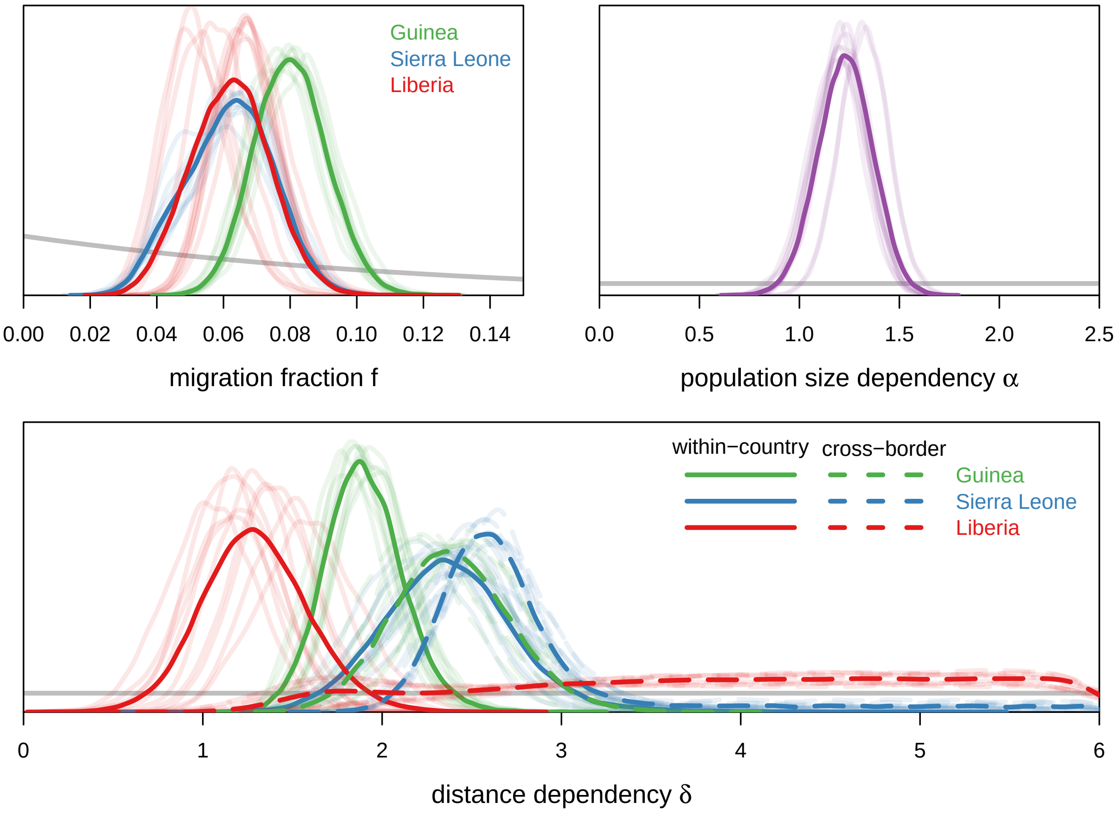

Figure B: Posterior distributions for model parameters: migration fraction f, population size dependency α and distance dependency for within-country (solid lines) and cross-border (dashed lines) transmissions, for Guinea (green), Sierra Leone (blue), Liberia (red) or all countries (purple), with prior distributions (grey). The transparent lines are the posterior distributions for ten augmented data sets, the non-transparent line is the grouped posterior distribution.

parameter		posterior values	
		median	95% CI
migration fraction Guinea	$f(G)$	0.0801	(0.0595 - 0.102)
migration fraction Sierra Leone	$f(S)$	0.0622	(0.0355 - 0.0864)
migration fraction Liberia	$f(L)$	0.0622	(0.0389 - 0.0857)
population size dependency	α	1.23	(0.953 - 1.50)
distance dependency within Guinea	$\delta(G, G)$	1.89	(1.49 - 2.36)
distance dependency within Sierra Leone	$\delta(S, S)$	2.36	(1.68 - 3.13)
distance dependency within Liberia	$\delta(L, L)$	1.29	(0.730 - 1.91)
distance dependency cross-border from Guinea	$\delta(G, .)$	2.38	(1.77 - 3.08)
distance dependency cross-border from Sierra Leone	$\delta(S, .)$	2.63	(2.12 - 5.40)
distance dependency cross-border from Liberia	$\delta(L, .)$	4.01	(1.54 - 5.90)

Table C: Posterior values of model parameters, estimated for 2014 ebola epidemic in West Africa: Guinea (G), Sierra Leone (S) and Liberia (L)

3 Survival analysis

To study whether countries differ in susceptibility of a district to be infected, we analyse the survival of districts till infection, as a function of the expected number of imported cases. This number $\kappa_i(t)$ to district i at time t is the sum of the contributions of all other districts:

$$\kappa_i(t) = \sum_{j \neq i} f(c_j) m_{j,i} \Lambda_j(t),$$

where $f(c)$ is the country-specific migration fraction, and the dispersion term $m_{j,i}$ and the local incidence $\Lambda_j(t)$ are defined as in the manuscript in equations 4 and 1. Figure C shows the expected number of imported cases $\kappa_i(t)$ in each district, as well as the moment(s) of infection. The shaded areas indicate the cumulative expected number of imported cases while the district was uninfected, and have size X_k. These areas end with an infection (denoted by an arrow in Fig. C), or not due to the end of the epidemic (censored data). Per country the number of observations n are ordered by increasing cumulative expected number of imported cases X_k with a label $d_k = 1$ for infection and $d_k = 0$ for escape. The number of observations n is 61 for Guinea, 24 for Sierra Leone, and 32 for Liberia.

The Kaplan-Meier estimate of the survival function S_k is calculated as:

$$S_k = \prod_{j \leq k} \left(1 - \frac{d_j}{n - j + 1}\right) \qquad \text{for} \quad 1 \leq k \leq n,$$

for which $(1 - \alpha)$ confidence intervals are based on Greenwood's estimator of variance:

$$S_k^{\text{upper}} = S_k + z_{1-\alpha/2} \sqrt{S_k^2 \sum_{j \leq k} \frac{d_j}{(n - j + 1)(n - j + 1 - d_j)}} \qquad \text{for} \quad 1 \leq k \leq n$$

$$S_k^{\text{lower}} = S_k - z_{1-\alpha/2} \sqrt{S_k^2 \sum_{j \leq k} \frac{d_j}{(n - j + 1)(n - j + 1 - d_j)}} \qquad \text{for} \quad 1 \leq k \leq n$$

Plotting survival function S versus cumulative expected number of imported cases X yields the survival plots in Figure D. There is no marked difference between the Kaplan-Meier survival curves for the different countries, which can be interpreted as the countries having a comparable district susceptibility to infection.

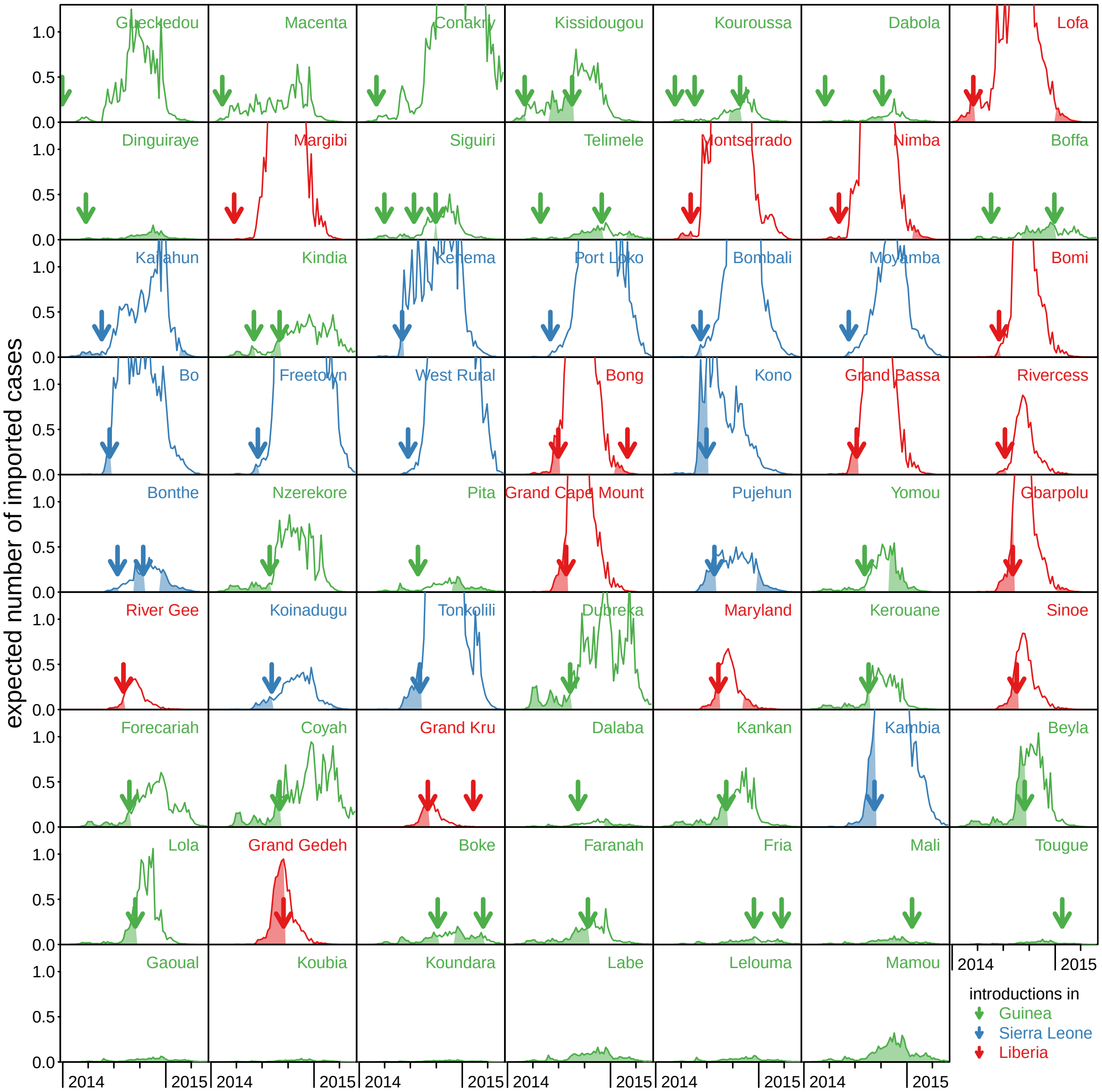

Figure C: The expected number of imported cases in each district in Guinea (green), Sierra Leone (blue) and Liberia (red), based on median posterior values, averaged over ten augmented data sets. Districts are ordered by time of first observed case. Arrows indicate the moment(s) of first observed case, and the shaded areas indicate when the district was uninfected (again). Note that not all districts are infected (e.g. Mamou, Guinea) and some districts are infected multiple times (e.g. Siguiri, Guinea).

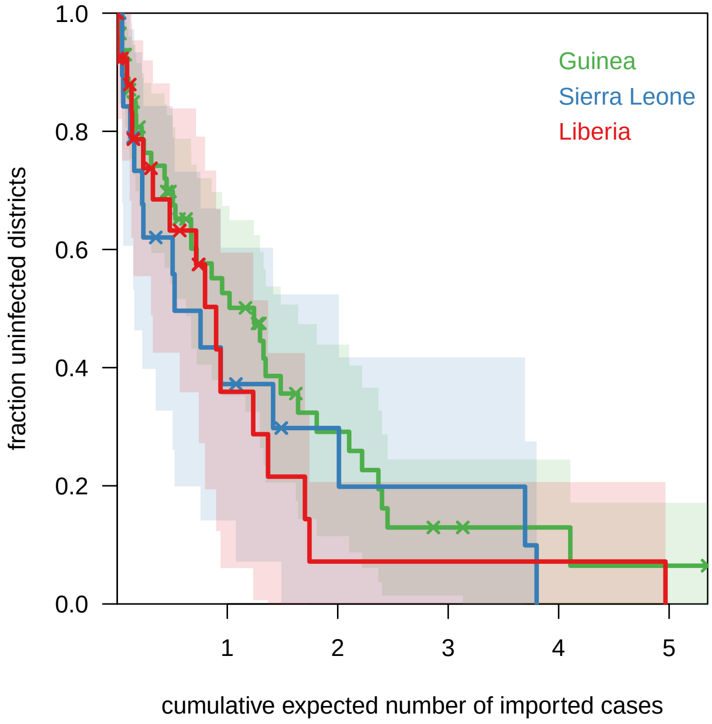

Figure D: Kaplan-Meier survival curves for uninfected districts in Guinea (green), Sierra Leone (blue) and Liberia (red). Survival is taken as a function of the cumulative expected number of imported cases; 95% confidence intervals are shown in transparent colours, censoring (i.e. districts escaping infection) is denoted by crosses.

4 Sensitivity analysis underreporting

To explore the effect of assuming perfect reporting, the analysis is repeated for one data set with different levels of underreporting in Guinea. The underreporting fraction is varied between 0% (perfect reporting) to 50% (actual number of cases is twice the number of reported cases). The parameter posteriors (Fig. E) become more variable with increasing underreporting, but do not seem to increase or decrease by much. A notable exception is the migration fraction in Guinea, that decreases from 8.1% to 6.7% on average. With the increasing number of cases, relatively less cases need to migrate to account for the between-district transmissions, while the absolute number of migrating cases does increase (Fig. 5 in main text). At the same time, this larger number of cases in Guinea can be held responsible for more of the (re)introductions in Guinean districts, diminishing the role of Sierra Leone in this respect (Fig. F).

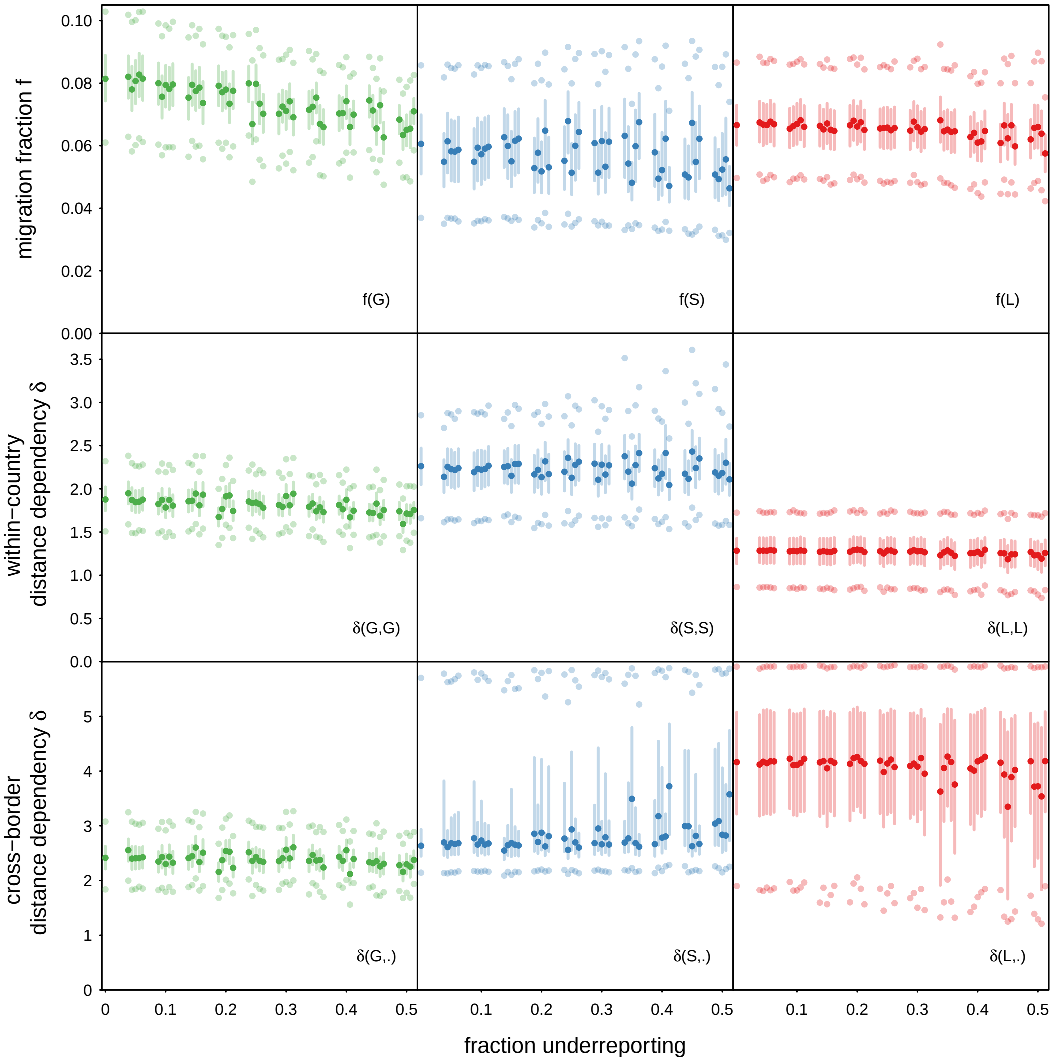

Figure E: Posterior parameter distributions as a function of underreporting fraction in Guinea, with five repetitions per underreporting level, for Guinea (green), Sierra Leone (blue) and Liberia (red). Median value (dark symbol), interquartile range (light line), and 2.5% and 97.5% percentiles (light symbols); parameter symbols as defined in Tab. C.

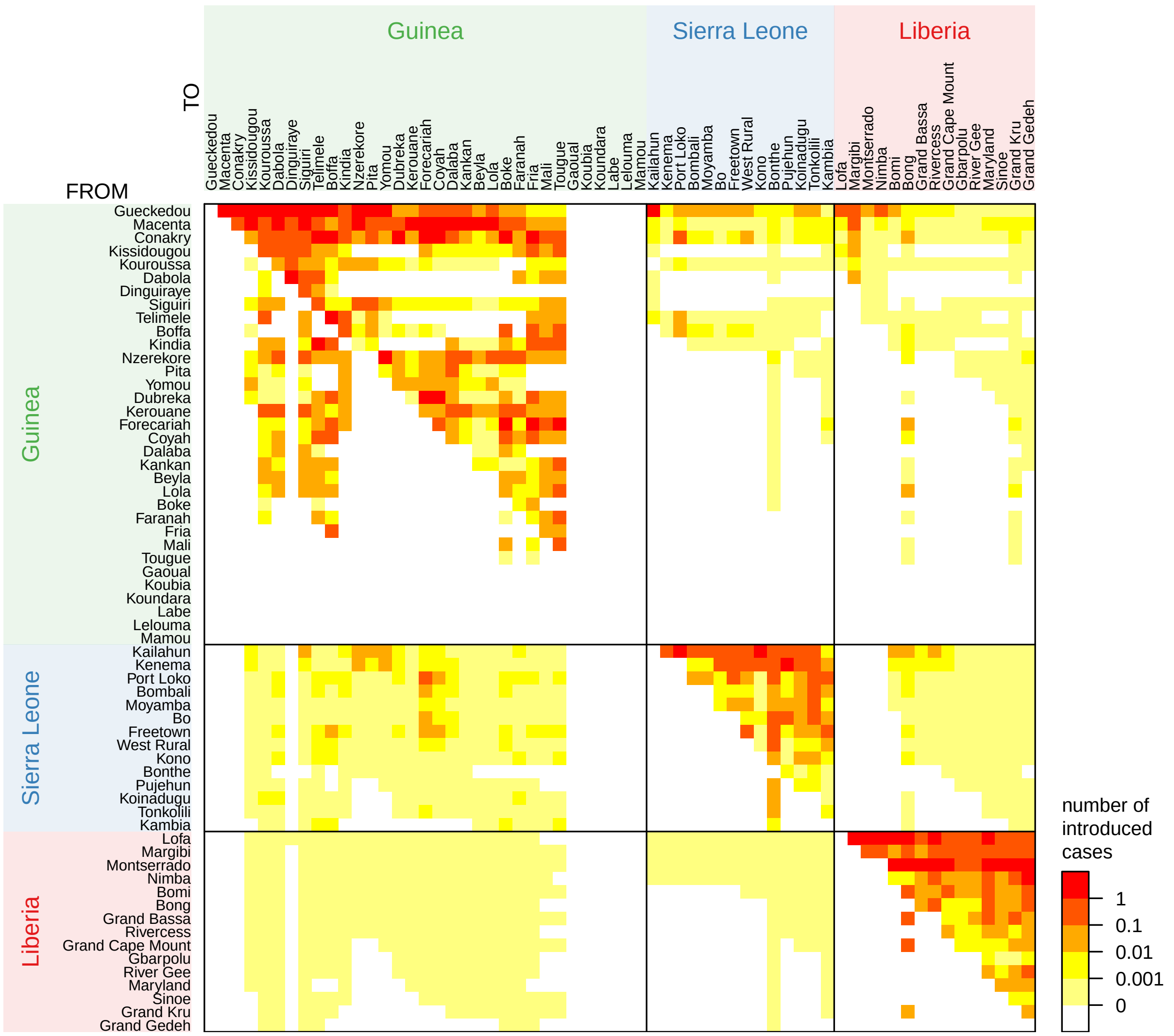

Figure F: Number of introduced cases per district distributed over possible origin districts, based on median posterior values averaged over five repetitions for **underreporting fraction of 0.50**; to be compared to the analysis without underreporting (Fig. 4 in main text). Districts are ordered per country by time of first observed case. District columns add up to the total number of observed introduced cases in that district, which can be higher than 1 due to multiple introductions and due to multiple cases per introduction. Colours indicate distinct categories: 1 or more introduced cases (red), between 0.1 and 1 (dark orange), between 0.01 and 0.1 (orange), between 0.001 and 0.01 (yellow), between 0 and 0.001 (light yellow), and 0 (white). The latter category means that this introduction is impossible, because the destination was never infected or the source was not infected at the time of introduction in the destination.

5 Sensitivity analysis serial interval

To explore the effect of the assumed serial interval, the analysis is repeated for one data set with different average serial interval. The shape parameter of the gamma distribution is fixed at the original value of 2.7 for all analyses. Most estimated parameters are constant over the range of serial intervals, except for the migration fractions in Sierra Leone and Liberia (Fig. G). With a shorter average serial interval, these fractions are found to be higher which leads to more migrating cases. In Sierra Leone, a large part of these additional migrations leave for other countries, while in Liberia, they stay within the country (Fig. H). The distribution of origin districts for (re)introductions also shows the enhanced role of Sierra Leone in cross-border transmissions for a short average serial interval (Fig. I), although these results should not be overinterpreted with the sensitivity analysis results for underreporting in mind (section 4).

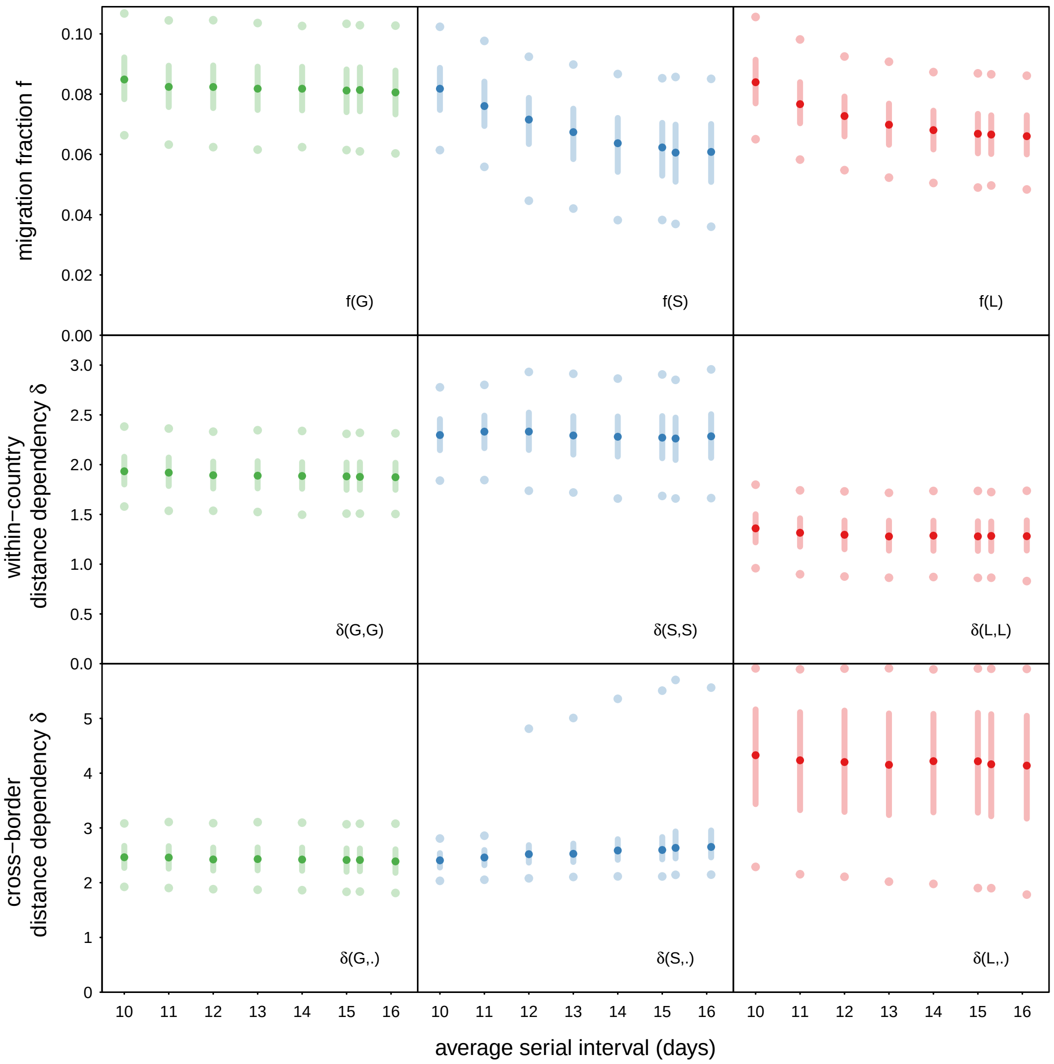

Figure G: Posterior parameter distributions as a function of average serial interval, for Guinea (green), Sierra Leone (blue) and Liberia (red). The average serial interval used in the main analysis is 15.3 days. Median value (dark symbol), interquartile range (light line), and 2.5% and 97.5% percentiles (light symbols); parameter symbols as defined in Tab. C.

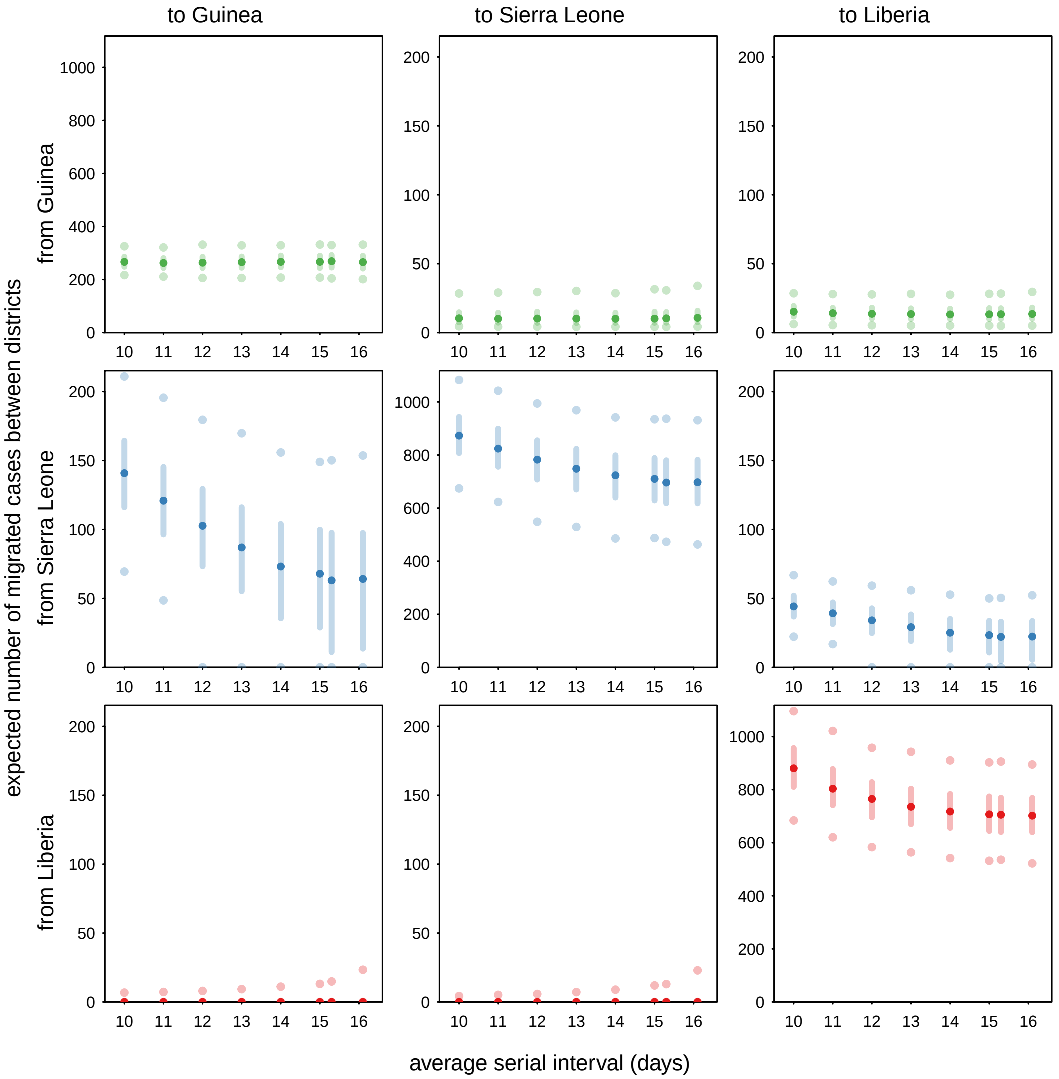

Figure H: Expected number of migrated cases between districts as a function of average serial interval, based on median posterior values, for Guinea (green), Sierra Leone (blue) and Liberia (red). The average serial interval used in the main analysis is 15.3 days. Median value (dark symbol), interquartile range (light line), and 2.5% and 97.5% percentiles (light symbols).

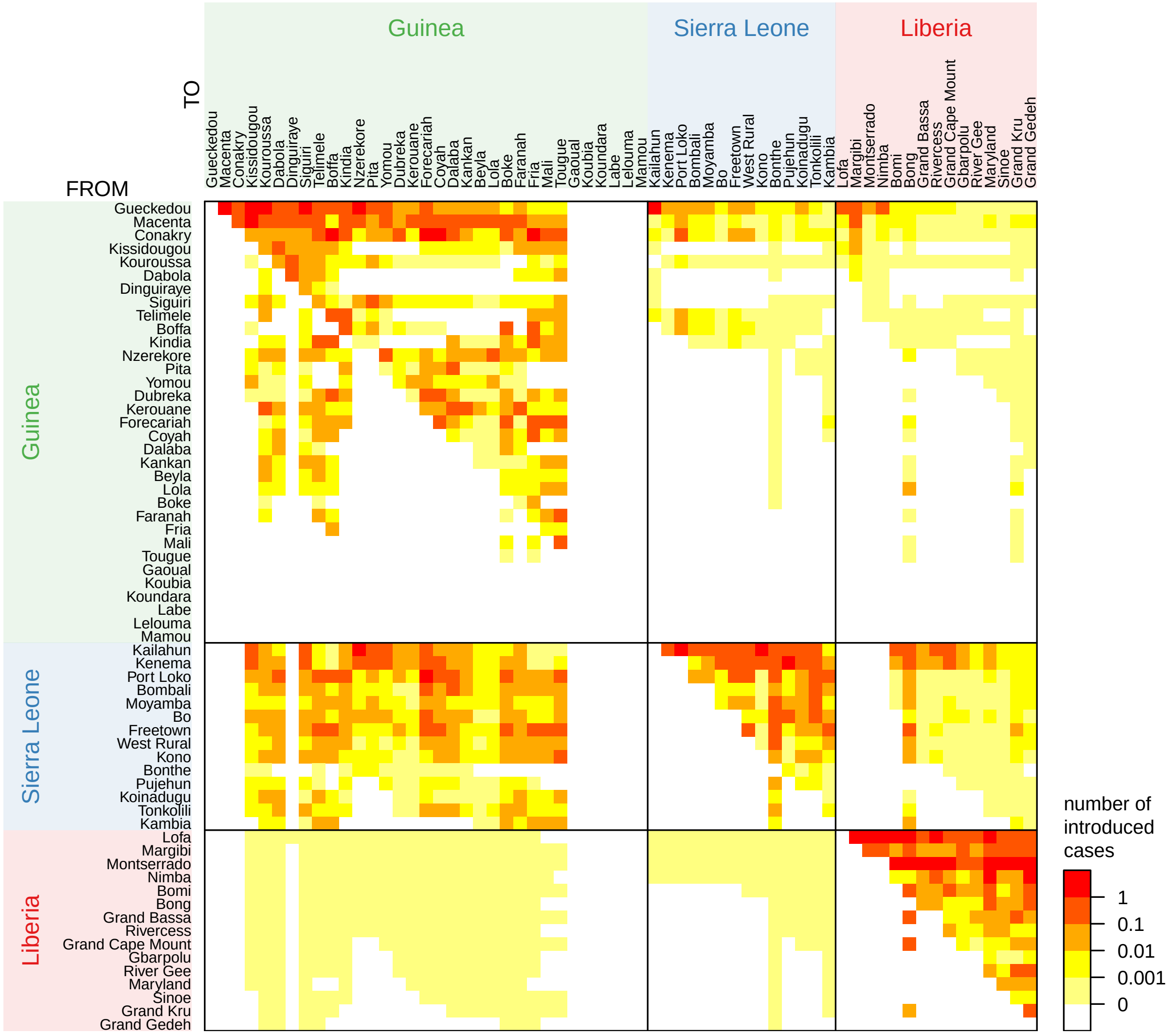

Figure I: Number of introduced cases per district distributed over possible origin districts, based on median posterior values for **average serial interval of 10 days**; to be compared to the analysis with an average serial interval of 15.3 days (Fig. 4 in main text). Districts are ordered per country by time of first observed case. District columns add up to the total number of observed introduced cases in that district, which can be higher than 1 due to multiple introductions and due to multiple cases per introduction. Colours indicate distinct categories: 1 or more introduced cases (red), between 0.1 and 1 (dark orange), between 0.01 and 0.1 (orange), between 0.001 and 0.01 (yellow), between 0 and 0.001 (light yellow), and 0 (white). The latter category means that this introduction is impossible, because the destination was never infected or the source was not infected at the time of introduction in the destination.

6 Analysis with time-dependent parameters

To explore the effect of assuming time-independent parameters, the analysis is repeated for one of the augmented data sets (that best resembles the average) with two defined control phases per country. The change points of these phases are chosen to reflect the transition from little control measures to maximal control. These points in time are by no means straightforward to determine and differ from district to district. As a proxy, we choose 1 August 2014 for Guinea (declaration of public health emergency of international concern by the WHO), 21 August 2014 for Liberia (border closures and West Point quarantine) and 21 September 2014 for Sierra Leone (nationwide 72-hour lockdown).

The migration fractions f and the distance dependencies δ are estimated for the 'after' and 'before' control phase of their respective countries (Fig. J). For all countries, the migration fraction decreases after the change point, but less so for Guinea. The within-country spatial dispersion becomes more local or stays the same (higher or comparable δ-values). The most probable explanation for these results is that most districts are infected in the early stages of the epidemic, rather than the effect of control measures. Because transmission was still ongoing in Guinea during the later stages, the migration fraction in Guinea stays relatively high in the second control phase. Cross-border transmissions only play a role in the first control phase in Guinea and Sierra Leone. For all other phases, the data contain no information, as is apparent from the 'cigar shapes' that reflect the prior distribution.

Similarly to the sensitivity analyses for underreporting and the serial interval (sections 4 and 5), the effect on the number of migrating cases within and between countries is assessed (Tab. D). Within countries, less of these case migrations take place, due to the more local character of transmissions in the second control phase. The numbers of cross-border transmissions, however, are comparable in both cases, as most of these take place in the first control phase. This is also clear from the distribution of possible origin districts for (re)introductions (Fig. K). Sierra Leone seems to contribute considerably to cross-border introductions in the first control phase up to the first observed case in Kankan in Guinea, but afterwards its contribution is negligible.

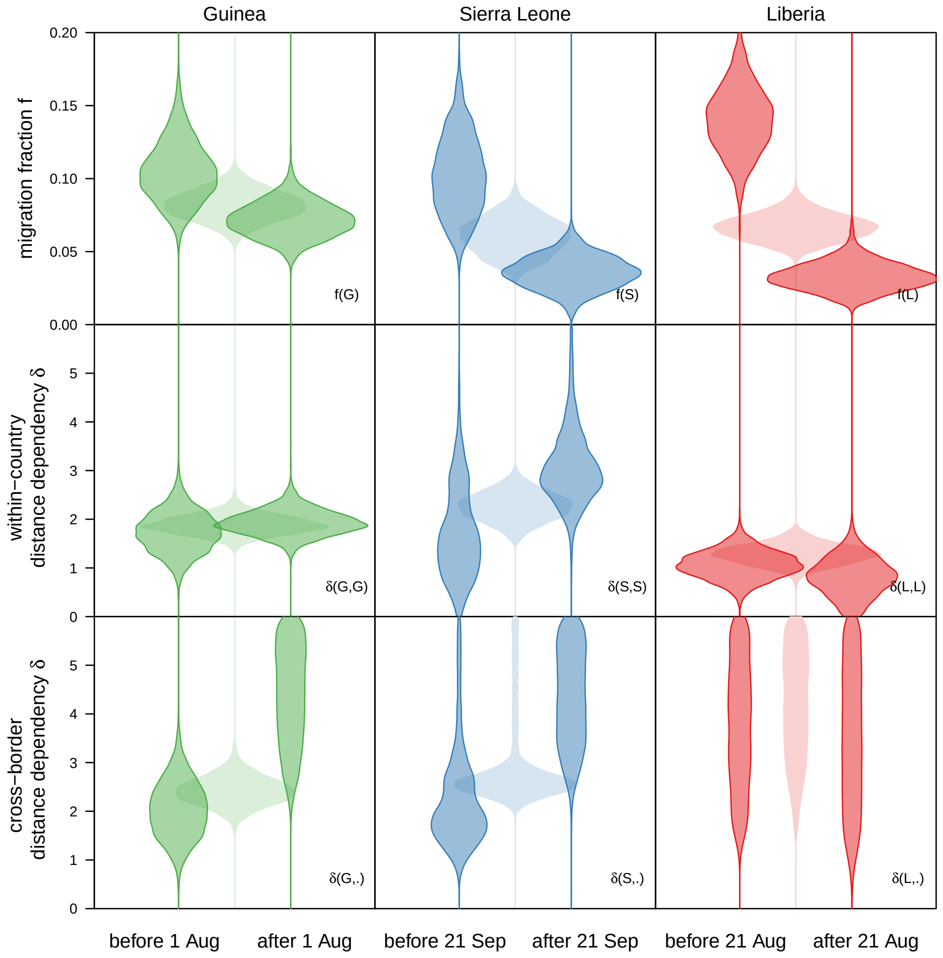

Figure J: Violin plots of posterior parameter distributions for analysis with two control phases, for Guinea (green, change point at 1 August 2014), Sierra Leone (blue, change point at 21 September 2014) and Liberia (red, change point at 21 August 2014). The posterior distributions for the original analysis with time-independent parameters are shown in the background (light shaded violin plots); parameter symbols as defined in Tab. C.

Expected number of migrated cases between districts	parameters time-dependent	parameters time-independent
within Guinea	254 (191 - 327)	269 (204 - 330)
within Sierra Leone	524 (301 - 760)	696 (473 - 937)
within Liberia	540 (370 - 744)	705 (536 - 906)
from Guinea to Sierra Leone	6.0 (3.4 - 17)	10 (4.2 - 31)
from Guinea to Liberia	13 (5.8 - 22)	13 (4.9 - 28)
from Sierra Leone to Guinea	42 ($5.0 \cdot 10^{-3}$ - 126)	63 ($1.1 \cdot 10^{-5}$ - 150)
from Sierra Leone to Liberia	28 ($2.1 \cdot 10^{-3}$ - 118)	22 ($2.8 \cdot 10^{-5}$ - 50)
from Liberia to Guinea	0.14 ($4.4 \cdot 10^{-5}$ - 27)	0.011 ($2.1 \cdot 10^{-5}$ - 15)
from Liberia to Sierra Leone	0.059 ($4.2 \cdot 10^{-6}$ - 29)	0.0020 ($1.5 \cdot 10^{-6}$ - 13)

Table D: Expected number of migrated cases within and between countries, estimated with time-dependent (2 control phases) and time-independent parameters.

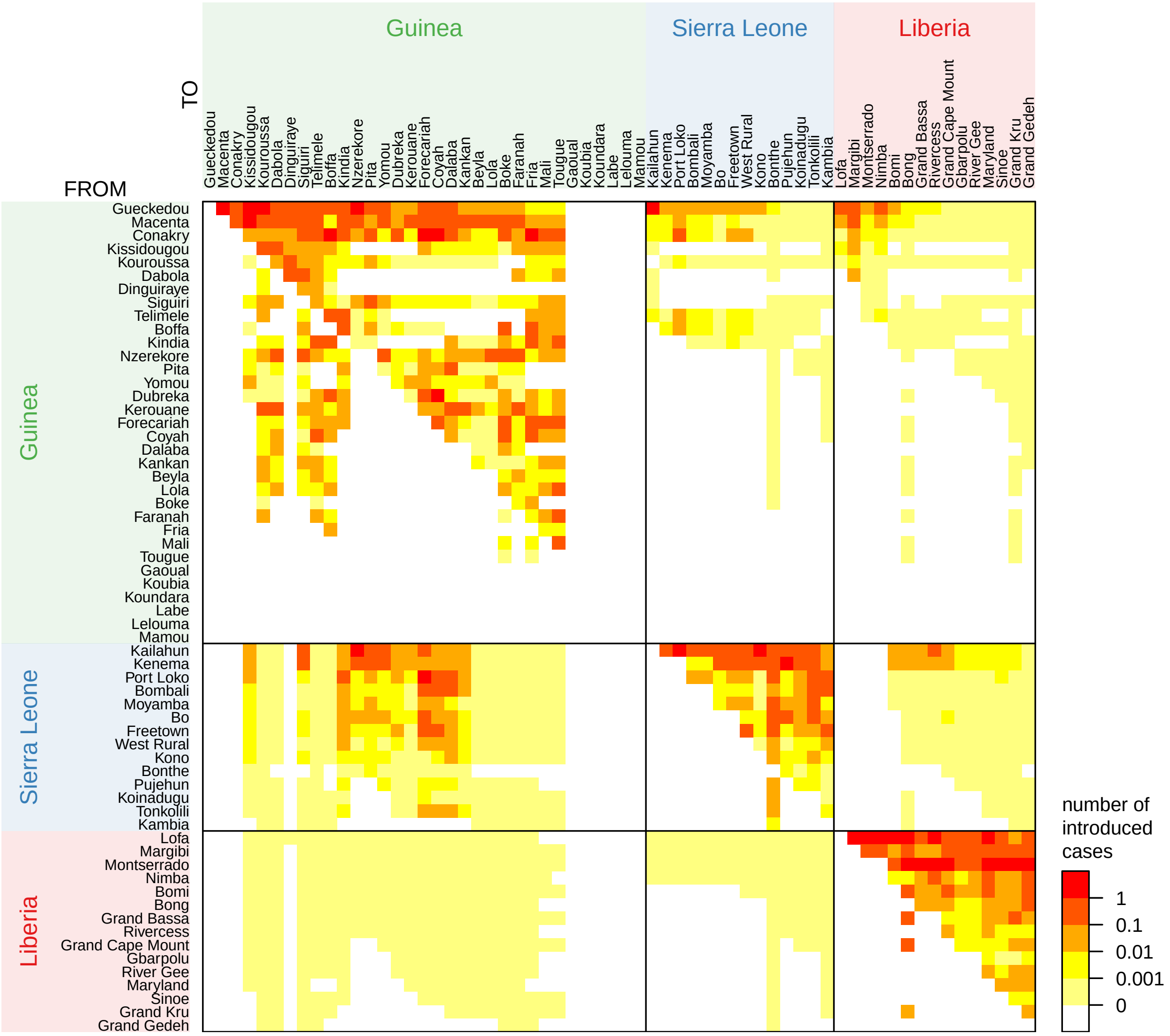

Figure K: Number of introduced cases per district distributed over possible origin districts, based on median posterior values of **time-dependent parameters**; to be compared to the analysis with time-independent parameters (Fig. 4 in main text). Districts are ordered per country by time of first observed case. District columns add up to the total number of observed introduced cases in that district, which can be higher than 1 due to multiple introductions and due to multiple cases per introduction. Colours indicate distinct categories: 1 or more introduced cases (red), between 0.1 and 1 (dark orange), between 0.01 and 0.1 (orange), between 0.001 and 0.01 (yellow), between 0 and 0.001 (light yellow), and 0 (white). The latter category means that this introduction is impossible, because the destination was never infected or the source was not infected at the time of introduction in the destination.
